# Anthelmintic resistance among gastrointestinal nematodes of cattle on dairy calf to beef farms in Ireland

**DOI:** 10.1186/s13620-020-00167-x

**Published:** 2020-07-01

**Authors:** Anne C. Kelleher, Barbara Good, Theo de Waal, Orla M. Keane

**Affiliations:** 1 Animal & Bioscience Department, Teagasc Grange, Dunsany, Co. Meath Ireland; 2 School of Veterinary Medicine, University College Dublin, Belfield, Dublin 4 Ireland; 3 Animal & Bioscience Department, Teagasc Mellows Campus, Athenry, Co. Galway Ireland

**Keywords:** Anthelmintic resistance, Gastrointestinal nematodes, Cattle, *Cooperia*, *Ostertagia*

## Abstract

**Background:**

The control of gastrointestinal nematodes (GIN) of cattle in pasture-based production systems such as Ireland is highly dependent on the availability of efficacious anthelmintics. There is very little information available on the efficacy of the broad-spectrum anthelmintics against GIN of cattle in Ireland and the aim of this study was to determine the prevalence of anthelmintic resistance on dairy calf to beef farms.

**Results:**

GIN burden was monitored on thirty-six recruited farms by performing herd level faecal egg counts (FEC) every 2 weeks. Of these, nine farms were lost from the study as calves were treated with an anthelmintic for *Dictyocaulus viviparus*, two were lost as they treated for GIN, one dropped out of the study and on one the herd FEC did not reach the threshold for carrying out the Faecal Egg Count Reduction Test (FECRT). On the remaining 23 farms, once the herd FEC reached 100 eggs per gram, a FECRT was carried out. Pre and post-treatment larval cultures were also performed to identify the GIN to genus level. The efficacy of fenbendazole, levamisole, ivermectin and moxidectin was evaluated on 15, 11, 16 and 11 farms respectively. Resistance to fenbendazole was identified on 9 farms (60%) with resistance suspected on a further farm. Resistance to levamisole, ivermectin and moxidectin was detected on 2 (18%), 16 (100%) and 8 (73%) farms respectively. The predominant genera detected pre and post-treatment were *Cooperia* and *Ostertagia* with both genera detected post-treatment with fenbendazole and ivermectin. Due to the low proportion of *Ostertagia* spp. pre-treatment, the efficacy of levamisole or moxidectin against this genus could not be reliably established.

**Conclusions:**

Anthelmintic resistance was widespread on the sampled dairy calf to beef farms in Ireland with resistance to benzimidazole, levamisole, ivermectin and moxidectin detected.

## Background

Irish beef production is predominately grass-based, with farmers aiming to have calves out to pasture as early as possible in the grazing season. However, an inevitable consequence of grazing is infection with nematode parasites, such as the gastrointestinal nematodes (GIN) *Cooperia* spp. and *Ostertagia* spp. and the lungworm *Dictyocaulus viviparus* [1, 2]. Calves in their first grazing season (FGS) are most at risk from these nematodes as they have not yet developed immunity [3, 4]. Heavy infestations of these nematodes can cause substantial economic losses in young calves due to ill-thrift, in addition to morbidity and sometimes even mortality [4, 5]. Anthelmintic treatments are often administered either prophylactically to prevent such losses or therapeutically to treat nematode infections [6]. The availability of efficacious anthelmintic products is therefore of great importance in Irish cattle rearing systems.

There are currently three classes of broad-spectrum anthelmintics available for the control of GIN in cattle in Ireland, benzimidazoles (BZ), imidazothiazoles (LV) and macrocyclic lactones (ML). However, the chemoprophylactic approach to GIN control is threatened by the emergence of anthelmintic resistant nematode populations [7]. Anthelmintic resistance (AR) among GIN of small ruminants has previously been described [8–10] with widespread resistance reported in Ireland including populations of multi-drug resistant *Teladorsagia circumcincta* [11–13]. AR in GIN of cattle has been reported less frequently, although resistance has been identified in New Zealand, Australia, Europe and the USA [7, 14, 15]. While initial reports of inefficacy of these drugs identified the dose-limiting *Cooperia* spp. as the major species found post-treatment, inefficacy against *Ostertagia* spp. is increasingly reported [16–18]. Despite these reports, there is a dearth of knowledge regarding the extent of AR on Irish cattle farms. One study examined AR on 2 Irish beef research farms, enterprises with a large number of animal movements. On one farm fenbendazole, levamisole (LV) and ivermectin (IVM) were tested while on the second farm only IVM was tested. On both farms IVM resistant *Cooperia* spp. were identified [19]. A further study carried out on 4 dairy farms in the East of Ireland identified IVM resistant *Cooperia* spp. on each of them and resistant *Ostertagia* spp. on one farm [20]. Therefore, there is a need to quantify the extent of AR on cattle farms in Ireland. The aim of this study was to determine the efficacy of the three classes of anthelmintic drugs on commercial dairy calf to beef farms from Ireland.

## Methods

### Recruitment of farms

The study took place over the summers of 2017 and 2018. Farmers were recruited via the Teagasc drystock advisory service and interested farmers self-selected. In order to be considered for inclusion in the study, farmers required good animal handling facilities and to agree to submit calf faecal samples every 2 weeks until the faecal egg count reduction test (FECRT) was conducted. A minimum of 40 FGS calves was preferred. No attempt was made to ensure a systematic survey. Thirty-six dairy calf to beef farmers signed up for the study; 20 in 2017 and 16 in 2018. Four farms that participated in 2017 also participated in 2018 resulting in 20 farmers participating each year.

### Herd faecal egg count monitoring

In order to monitor the herd faecal egg count (FEC), participating farmers were required to collect fresh field faecal samples from 10 to 15 FGS calves every 2 weeks from the 1st of May and submit the samples to Teagasc. Once the faecal samples were received, a composite sample was generated by pooling 5 g of faeces from each calf and mixing well. Nematode eggs in the composite faecal sample were enumerated using a modified mini-FLOTAC method with a sensitivity of 5 epg. In brief, 5 g of the composite sample was suspended in 45 ml of deionised water. Large debris was subsequently removed by passing the slurry solution through a 250 μm sieve (Endecotts); the flow-through was then centrifuged at 433 *g* for 3 min and the pellet resuspended up to 50 ml with saturated salt solution (specific gravity = 1.2). The solution was inverted three times to mix and immediately used to fill 2 chambers of a mini-FLOTAC disk [21]. Eggs present in both chambers that were identified as Strongylid eggs were enumerated. The remainder of the composite faecal sample was examined for lungworm larvae using the Baermann technique [22]. If lungworm larvae were detected, the farmer was immediately contacted and advised of the result and invariably the calves were treated with an anthelmintic. The farm was then removed from the study to ensure the test was not performed on a pre-selected population of GIN. Herd faecal sample collection continued until the FECRT was carried out or the farm was removed from the study.

### Faecal egg count reduction test

Once the herd FEC reached approximately 100 epg the farmer was contacted and a date arranged to visit the farm. A conservative 100 epg herd FEC threshold was chosen as it was considered sufficient to allow calculation of the egg count reduction after anthelmintic treatment but conservative enough that it would have been unlikely that some animals could have very high counts that could cause clinical disease in individual animals, which would have been unacceptable to the farmers. In 2017, BZ and IVM were tested and each farm was visited twice (days 0, 14). On day 0, up to 40 calves from the grazing group were selected and systematically allocated to one of two groups as they entered the crush. All calves were weighed using an electronic scale (Tru-Test) and individual faecal samples collected *per rectum* from each calf. One group of calves was treated with IVM (Ivomec, Merial Animal Health Ltd) subcutaneously at a rate of 0.2 mg/kg body weight. The second group of calves was treated with oral fenbendazole (Panacur, Intervet Ireland Ltd) at a rate of 7.5 mg/kg body weight. All anthelmintic delivery equipment was calibrated on the day of administration. The calves returned to pasture after treatment. Faecal samples were stored in 70 ml faeces tubes (Starstedt) and transported to the laboratory where they were stored at 4 °C until analysis. On day 14 post-treatment the farm was revisited and faecal samples again collected from the calves *per rectum*. In 2018, LV and moxidectin (MOX) were tested and each farm was visited 3 times (days 0, 7 and 14). On day 0, up to 40 calves from the grazing group were selected and systematically allocated to one of two groups as they entered the crush, all calves were weighted using an electronic scale and individual faecal samples collected *per rectum* from each calf. The first group received MOX (Cydectin, Zoetis Ireland Ltd) subcutaneously at a rate of 0.2 mg/kg body weight. The second group received oral LV (Levacide Low Volume Worm Drench, Norbrook Laboratories Limited) at a rate of 7.5 mg/kg body weight. All anthelmintic delivery equipment was calibrated on the day of administration. The calves returned to pasture after treatment. Individual calf faecal samples were collected *per rectum* from the calves that received LV on day 7 post-treatment while individual calf faecal samples were collected from calves that received MOX on day 14 post-treatment. On one farm in 2018, all 4 anthelmintics were tested. The FECRT was conducted on calves that had not previously been treated with an anthelmintic product; however, on one farm (farm 10) the calves were treated with a BZ product prior to the start of the study; 4 months before the FECRT was conducted.

Individual calf faecal samples were analysed for GIN FEC using the mini-FLOTAC method as described above. Only animals with both a pre and post-FEC were included and animals with a pre-treatment FEC < 10 epg were excluded from FECRT calculation. Groups with a mean pre-treatment FEC < 40 epg were also excluded from further analysis due to the low FEC. Data were analysed using the Shiny web interface for the R package eggCounts with paired samples and allowing individual efficacy [23]. If the mean FEC increased after anthelmintic treatment, the default prior for modelling the reduction was changed to Uniform(0,4) [23]. Resistance was considered present if the percentage reduction in egg count was < 95% and the lower value of the 95% uncertainty interval was < 90%. If only one of these two criteria was met then resistance was suspected [24].

### Larval identification

Larvae were cultured using standard techniques. Briefly, faeces were mixed with vermiculite and water and incubated at 23 °C for 14 days with the culture mixed daily for the first 3 days and water added as needed. Third-stage larvae were subsequently recovered by baermanisation [22]. A larval culture containing an equivalent mass of faeces from all animals with sufficient faecal material available was carried out for pre-treatment samples collected on day 0, while coprocultures for each treatment group were carried out with faeces collected on day 7 or day 14. Larvae were stored in 175 ml culture flasks with vented caps at 4 °C until larval identification. For each sample pre and post-treatment at least 100 L3 (or however many L3 were available if < 100) were identified to genus level according to the diagrams and keys of van Wyk and Mayhew [25]. The proportion of *Cooperia* spp. and *Ostertagia* spp. in each faecal culture was used to apportion the FEC pre and post anthelmintic treatment and to calculate the faecal egg count reduction (FECR) for each genus using a modified version of the RESO calculator, resoLootNew (https://wormmailinthecloud.wordpress.com/2011/03/25/drench-efficacy-calculators-update/). A mean pre-treatment FEC of 20 epg was considered sufficient to calculate the FECR for each genus.

## Results

### Participating farmers

In total 36 farmers were recruited to the study; 20 farmers enrolled in 2017 while in 2018 a further 16 farmers enrolled while 4 farmers that had taken part in 2017 re-enrolled. However, the number of FECRTs completed was 15 in 2017 and 12 in 2018. In 2017, 4 farms were lost as calves were treated with an anthelmintic (2 treated for *Dictyocaulus viviparus* and 2 treated for GIN) and one farmer dropped out of the study. In 2018, 7 farms were lost as calves were treated with an anthelmintic (all were treated for *Dictyocaulus viviparus*) while on one farm the herd FEC did not exceed 20 epg and so the FECRT was not attempted. In total 3 tests were excluded as pre-treatment FEC did not reach the threshold of 40 epg. The number of tests completed and available for analysis for each anthelmintic is shown in Table 1.

**Table 1:** Number of farms that completed a faecal egg count reduction test (FECRT) with each anthelmintic class and the number available for analysis after data quality control

No. farms enrolled	No. FECRT completed	No. FECRT available for analysis
**Benzimidazole**		
21	16	15
**Levamisole**		
20	12	11
**Ivermectin**		
21	16	16
**Moxidectin**		
20	12	11

### Faecal egg count reduction test

The efficacy of BZ was evaluated on 15 farms; of these, BZ resistance was identified on 9 farms with FECRs ranging from 27 to 94% (Table 2). BZ was determined to be effective against GIN on 5 farms, while resistance was suspected on one farm (Farm 1; FECR = 96, 95% U.I. = 86–100%). LV efficacy was evaluated on 11 farms; resistant GIN were identified on 2 farms with FECRs of 83 and 92% while GIN on the remaining 9 farms displayed LV susceptibility (Table 2). The efficacy of IVM and MOX was evaluated on 16 and 11 farms, respectively. Resistant GIN to IVM were confirmed on all 16 farms examined with FECRs ranging from − 181 - 93%; in contrast resistance to MOX was identified on 8 farms (FECRs 34–94%) with MOX susceptibility confirmed on 3 farms (Table 2).

**Table 2:** Number of animals, faecal egg count (FEC) pre and post treatment and FEC reduction (95% uncertainty interval) for faecal egg count reduction tests (FECRT) carried out with benzimidazole, levamisole, ivermectin and moxidectin on dairy calf to beef farms in Ireland

Farm	Benzimidazole (BZ)				Levamisole (LV)				Ivermectin (IVM)				Moxidectin (MOX)			
	No. calves	Mean FEC (epg)		%FECR (95% UI)	No. calves	Mean FEC (epg)		%FECR (95% UI)	No. calves	Mean FEC (epg)		%FECR (95% UI)	No. calves	Mean FEC (epg)		%FECR (95% UI)
		Pre	Post			Pre	Post			Pre	Post			Pre	Post	
1	16	139	6	96 (86–100)	NT	NT	NT	NT	18	140	49	65 (47–78)	NT	NT	NT	NT
2	19	182	56	69 (57–78)	19	109	9	92 (70–100)	20	141	392	−181 (− 241- -101)	18	179	110	38 (19–59)
3	19	341	25	93 (89–95)	NT	NT	NT	NT	20	380	162	58 (43–69)	NT	NT	NT	NT
4	NT	NT	NT	NT	17	52	1	98 (95–100)	NT	NT	NT	NT	18	66	0	100 (98–100)
5^a^	18	54	1	98 (94–99)	20	48	1	99 (96–100)	19	56	6	89 (81–95)	15	69	1	99 (96–100)
6	15	287	17	94 (82–99)	NT	NT	NT	NT	14	223	190	15 (−78–74)	NT	NT	NT	NT
7	20	258	6	98 (96–99)	NT	NT	NT	NT	19	297	179	39 (20–59)	NT	NT	NT	NT
8	20	217	71	68 (55–77)	18	409	1	100 (100–100)	19	205	58	72 (59–80)	19	317	37	88 (83–92)
9	NT	NT	NT	NT	18	266	0	100 (100–100)	NT	NT	NT	NT	18	144	54	62 (37–84)
10	19	266	108	60 (37–75)	NT	NT	NT	NT	19	419	277	34 (16–54)	NT	NT	NT	NT
11	13	118	85	27 (12–50)	NT	NT	NT	NT	14	82	6	93 (84–98)	NT	NT	NT	NT
12	17	132	14	89 (82–94)	NT	NT	NT	NT	19	142	65	54 (32–71)	NT	NT	NT	NT
13	18	165	18	89 (82–94)	NT	NT	NT	NT	19	153	83	45 (24–62)	NT	NT	NT	NT
14	NT	NT	NT	NT	18	67	0	100 (99–100)	NT	NT	NT	NT	13	81	5	94 (89–98)
15	NT	NT	NT	NT	NC	NC	NC	NC	NT	NT	NT	NT	17	48	2	97 (92–99)
16	18	572	21	96 (94–98)	NT	NT	NT	NT	20	524	156	70 (55–82)	NT	NT	NT	NT
17	NT	NT	NT	NT	16	129	3	98 (95–100)	NT	NT	NT	NT	NC	NC	NC	NC
18	19	177	2	99 (98–100)	18	72	12	83 (72–91)	18	196	35	82 (74–88)	18	53	4	93 (86–98)
19	NT	NT	NT	NT	17	95	0	100 (96–100)	NT	NT	NT	NT	20	135	42	69 (52–80)
20	NT	NT	NT	NT	18	118	1	99 (97–100)	NT	NT	NT	NT	19	97	7	93 (87–97)
21	20	152	1	99 (98–100)	NT	NT	NT	NT	18	110	35	69 (50–82)	NT	NT	NT	NT
22	NC	NC	NC	NC	NT	NT	NT	NT	10	47	14	71 (45–86)	NT	NT	NT	NT
23	19	76	8	90 (84–95)	19	45	0	100 (98–100)	20	77	33	57 (34–74)	17	43	28	34 (17–62)

^a^FECRT with all 4 anthelmintic classes carried out in the same year. For all other farms, BZ and IVM were tested in 2017 and LV and MOX in 2018

*NC* not calculated: pre-treatment FEC < 40 epg

*NT* not tested: anthelmintic class not evaluated

### Larval identification

Larvae were recovered from coprocultures pre and post anthelmintic treatment for 12 farms in 2017 with the exception of Farm 21 post-IVM treatment and for all farms in 2018 with the exception of Farm 14 post-MOX treatment. *Cooperia* and *Ostertagia* were the predominant genera present on all farms, although on four farms a small number of other genera were present pre-treatment. Only *Cooperia* spp. or *Ostertagia* spp. were found post-treatment. The number of larvae, percentage of *Cooperia* spp. and *Ostertagia* spp. identified pre and post-anthelmintic treatment and anthelmintic efficacy against *Cooperia* spp. and *Ostertagia* spp. are shown in Tables 3 (BZ and IVM) and 4 (LV and MOX).

**Table 3:** Efficacy of benzimidazole (BZ) and ivermectin (IVM) against *Cooperia* spp. and *Ostertagia* spp. on Irish dairy calf to beef farms

Farm	Pre-treatment			Post-BZ treatment			Post-IVM treatment		
	No. L3	%***Cooperia***	%***Ostertagia***	No. L3	%***Cooperia*** (%efficacy)	%***Ostertagia*** (%efficacy)	No. L3	%***Cooperia*** (%efficacy)	%***Ostertagia*** (%efficacy)
2	100	67	33	100	3 (99)	97 (11)	100	76 (0)	24 (0)
3	100	32	68	100	13 (97)	87 (90)	100	96 (0)	4 (97)
5	100	41	59	N/A	N/A	N/A	100	96 (68)	4 (99)
6	100	76	24	100	48 (97)	52 (90)	98	81 (0)	17 (27)
7	100	4	96	N/A	N/A	N/A	100	33 (0)^a^	67 (54)
8	100	23	75	21	10 (84)	90 (56)	97	30 (65)	70 (75)
10	35	57	43	100	41 (63)	59 (30)	100	54 (7)	46 (40)
11	42	10	90	52	0 (100)^a^	100 (5)	100	44 (51)^a^	56 (93)
12	100	16	84	24	0 (100)	24 (87)	100	32 (0)	68 (58)
13	100	5	95	29	0 (100)^a^	100 (86)	100	84 (0)^a^	16 (91)
16	100	72	27	N/A	N/A	N/A	100	90 (51)	10 (85)
21	100	68	32	N/A	N/A	N/A	–	–	–

*N/A* not applicable: treatment was considered effective

^a^ Pre-treatment egg count apportioned to genus < 20 epg

- no larvae recovered

**Table 4:** Efficacy of levamisole (LV) and moxidectin (MOX) against *Cooperia* spp. and *Ostertagia* spp. on Irish dairy calf to beef farms

Farm	Pre-treatment			Post-LV treatment			Post-MOX treatment		
	No. L3	%***Cooperia***	%***Ostertagia***	No. L3	%***Cooperia*** (%efficacy)	%***Ostertagia*** (%efficacy)	No. L3	%***Cooperia*** (%efficacy)	%***Ostertagia*** (%efficacy)
2	100	100	0	100	67 (87)	33 (0)^a^	100	95 (46)	5 (0)^a^
4	100	76	24	N/A	N/A	N/A	N/A	N/A	N/A
5	100	41	59	N/A	N/A	N/A	N/A	N/A	N/A
8	100	95	3	N/A	N/A	N/A	100	99 (86)	1 (96)^a^
9	100	99	1	N/A	N/A	N/A	100	100 (48)	0 (100)^a^
14	100	87	13	N/A	N/A	N/A	–	–	–
15	100	22	87	NC	NC	NC	N/A	N/A	N/A
17	100	78	22	N/A	N/A	N/A	NC	NC	NC
18	100	97	3	100	91 (80)	9 (37)^a^	100	100 (92)	0 (100)^a^
19	66	100	0	N/A	N/A	N/A	100	100 (67)	0 (ND)^a^
20	100	95	5	N/A	N/A	N/A	100	100 (92)	0 (100)^a^
23	100	100	0	N/A	N/A	N/A	100	100 (5)	0 (ND)^a^

*N/A* not applicable: treatment was considered effective

*NC* not calculated: pre-treatment FEC < 40 epg

*ND* not determined: efficacy could not be determined as the genus was not detected pre-treatment

- no larvae recovered

^a^ Pre-treatment egg count apportioned to genus < 20 epg

## Discussion

There are over 92,000 farms in Ireland, of which over 54,000 are classed as cattle rearing and fattening enterprises, which includes both dairy calf to beef and suckler beef production [26]. Despite the importance of this sector to the agricultural industry in Ireland, and the industry’s reliance on a grass-based production system, there is a distinct lack of information on the prevalence of AR among GIN of cattle in Ireland, although resistance to IVM has been reported [19, 20]. This study provides clear evidence for resistance to all commonly available anthelmintic classes among GIN of cattle in Ireland, including resistance to IVM in *Ostertagia* spp. The study was carried out on dairy calf to beef farms throughout Ireland, although the majority were located in the Midlands, South and South East of the country where the dairy calf to beef industry is concentrated. The prevalence of anthelmintic treatment failure on sheep farms in Ireland does not depend on geographical region [12]; given the similar cattle production system throughout the country and large number of animal movements [27, 28], it is similarly expected that geographical region will have no impact on prevalence of AR on Irish cattle farms. The study farms had an average farm size of 48 ha, compared to the average for Irish cattle rearing farms of 31 ha and for other cattle farms excluding dairy (primarily cattle finishing enterprises) of 37 ha [26] indicating that these farms were larger than the average beef farming enterprise. Additionally, it must be noted that participating farmers self-selected; therefore these farmers may have been more interested in AR or may have perceived GIN control to be an issue on their farm. Therefore, these farms may not be truly representative of all dairy calf to beef farmers in Ireland. Notwithstanding this, other studies have confirmed that IVM resistance is common on cattle farms in Ireland, albeit few farms have been previously tested [19, 20]. Prior to this study, testing for AR had only occurred on one farm although some farmers reported that they perceived IVM had become less effective. Farm 5 had tested for and confirmed resistance to IVM 5 years prior to this study. Historically this farm treated all calves each year with ivermectin at 3, 8 and 13 weeks post-turnout. In general, anthelmintic treatment practices varied both within farms from year to year and between farms and almost all treated with anthelmintics for *Dictyocaulus*.

A number of studies have demonstrated AR in GIN of cattle worldwide [29–32]. In particular, for IVM, resistance is commonly found in *Cooperia* spp., which is the dose-limiting species [18]. *Ostertagia* spp. have been reported present after treatment with IVM in only a few studies [17, 33, 34], suggesting resistance to ML is not common for this genus. In this study, IVM resistance was detected on all farms (100%) tested. For all farms for which data was available, the reduction in the *Cooperia*-apportioned egg count after IVM treatment was < 95% while the reduction in *Ostertagia*-apportioned egg count after IVM treatment was < 95% on 9 farms and > 95% on 2 farms, indicating IVM resistance in both genera. On a number of farms, IVM failed to reduce the egg count apportioned to *Cooperia* at all. On one farm IVM-resistant but MOX-susceptible GIN were identified, a situation reported previously [13, 15]. MOX-resistance was also confirmed on 8/11 (73%) of farms tested. Resistance among *Cooperia* spp. to MOX was also demonstrated; on all farms with confirmed MOX resistance, the reduction in *Cooperia*-apportioned egg count was < 95%. Unfortunately the efficacy of MOX against *Ostertagia* spp. could not be tested due to the low abundance of *Ostertagia* in 2018.

BZ-resistance was also common, confirmed on 9/15 (60%) farms tested with a further farm showing suspected resistance. The efficacy of BZ against both *Ostertagia* spp. and *Cooperia* spp. was < 95% on some farms, indicating BZ resistance in both genera in Ireland. The most efficacious class of anthelmintic was LV, only 2 farms (18%) displayed LV resistance. On both of these farms efficacy against *Cooperia* spp. was < 95%; however efficacy against *Ostertagia* spp. could not be tested due to the low abundance of this genus. Generally *Cooperia* spp. are considered less pathogenic than *Ostertagia* spp. and calves are expected to develop immunity to *Cooperia* spp. reasonably quickly [35]. The detection of both ML and BZ-resistant *Ostertagia* spp. in Ireland is a particular cause for concern. LV has been reported to have poor efficacy against all stages of *O. ostertagia* [36] and BZ and ML anthelmintics are the most commonly recommended classes to use for the prevention of type II ostertagiosis due to their efficacy against both mature and immature nematodes [37].

The study was carried out in 2017 and 2018 but weather conditions for GIN transmission were more favourable in 2017. A drought in the summer of 2018 [38] meant that most farms presented with low herd FEC until at least mid-July. This was likely due to the lack of moisture, making it difficult for larvae to migrate out of the faecal pats combined with exposure of L3 to UV irradiation [39]. Therefore, mean FEC pre-treatment was generally higher in 2017 than in 2018. Notably, the 2018 weather conditions may also have differentially impacted *Ostertagia* spp. compared to *Cooperia* spp. as the proportion of *Ostertagia* spp. recovered from larval cultures was very low in 2018. This fact, combined with the lower pre-treatment FEC, meant that the proportion of FEC that could be apportioned to *Ostertagia* was too low to reliably test anthelmintic efficacy against this genus in 2018. However, it must be noted that many factors such as egg-laying capacity, immunity or density-dependent suppression of egg laying as well as the culture conditions, which may not be optimal for all species, can all have an effect on the recovery rate of the different GIN species. Therefore, the species-specific values determined from larval cultures may not necessarily fully reflect the true species-specific egg counts or the worm burden in vivo [40–42].

Putative infection with *D. viviparus* was another factor which limited the number of farms included in the study. The high prevalence of this nematode in Ireland [43] combined with the unpredictable nature and severe consequences of the disease in naïve calves means that farmers monitor calves closely and commonly treat with an anthelmintic at signs of respiratory disease [44]. In this study, 25% of participating farmers were excluded as calves were treated with an anthelmintic before herd FEC reached the 100 epg threshold required to carry out the FECRT. While a vaccine for *Dictyocaulus* is available in Ireland, uptake is low (MSD Ireland, pers. comm.) and the recommendation to house calves until at least 14 weeks of age is not compatible with optimising the contribution of grazed grass to feed intake [45]. A modified vaccine schedule in which calves were vaccinated at 6 and 8 weeks of age and turned out to pasture immediately after the second vaccination showed efficacy under delayed *Dictyocaulus* challenge; however, it was not as effective as the conventional vaccine schedule during severe pasture challenge [46]. The heavy reliance on anthelmintic drugs, particularly MLs, to control and treat *Dictyocaulus* infection may be a contributing factor in the rise of AR in GIN.

The use of an untreated control group is generally recommended for FECRT, in order to allow for natural changes in FEC during the test [47, 48]. In this instance, a control group was not utilised due to farmer’s perceptions of the risk from GIN and *Dictyocaulus* in an untreated group. However, the “eggCounts” package allowing individual efficacy was utilised along with the mini-FLOTAC method which has a sensitivity of 5 epg. Utilising an egg counting technique with high sensitivity has been reported to give good precision in the detection of AR [49]. It is also recommended for cattle that only oral formulations should be used in a FECRT [47]. Indeed it has been reported that oral MOX treatments are significantly less variable and more effective than injectable or pour-on treatments [50]. However, there is no oral IVM or MOX available on the market for cattle in Ireland.

The level of AR identified in this study demonstrates that strategies to manage GIN in the face of AR are urgently needed on Irish cattle farms. There is a dearth of information on parasite control practices on Irish cattle rearing and fattening farms and these need to be examined in order to identify risk factors associated with the development of AR and to take steps to mitigate these risks. Promoting the use of sustainable parasite control strategies is now imperative. Effective anthelmintic administration should be immediately promoted including appropriate anthelmintic selection, treating to the weight of the heaviest animal, calibration of anthelmintic administration equipment and good anthelmintic administration technique [51]. Strategies to increase refugia such as evaluating the need to treat older animals and grazing management, including avoiding moving stock to lowly contaminated pasture after treatment, should also be adopted. Indicators such as FEC or weight gain should be utilised to help target anthelmintic treatments [52]. In addition, further research on novel strategies for managing AR GIN or slowing the further development of AR, such as combination therapy or targeted selective treatment, is required. However, 43% of cattle rearing enterprises in Ireland are considered to be economically vulnerable, with the farm business not viable and no off-farm income [26]. Therefore strategies need to be developed that are appropriate to the needs and resources of Irish beef farmers.

## Conclusion

Irish cattle rearing relies on a pasture-based production system, aiming to maximise the contribution of grazed grass in the animal’s diet [28]. Effective nematode control is critical in this system and to-date such control has been dependent on the availability of efficacious anthelmintics. The high prevalence of anthelmintic resistance found by this study indicates that anthelmintic resistance is a threat to our grass-based production system. Sustainable worm control strategies, such as grassland management and targeted treatments, which prolong the life span of the currently available anthelmintics need to be implemented as a matter of urgency. However, as the emphasis in GIN control shifts from that of chemoprophylaxis, the impact on infection with *Dictyocaulus* will also need to be considered.

## Data Availability

The datasets analysed during the current study are available from the corresponding author on reasonable request.

## Abbreviations

AR: Anthelmintic resistance
BZ: Benzimidazoles
FEC: Faecal Egg Count
FECR: Faecal egg count reduction
FECRT: Faecal Egg Count Reduction Test
FGS: First grazing season
GIN: Gastrointestinal nematode
IVM: Ivermectin
LV: Levamisole
ML: Macrocyclic lactone
MOX: moxidectin
